# Blood proteomics: insights from public data

**DOI:** 10.1186/s13059-026-04027-9

**Published:** 2026-03-12

**Authors:** Asier Larrea-Sebal, Chengxin Dai, Alejandro J. Brenes, Kathrin Korff, Benjamin A. Neely, Philipp E. Geyer, Laura F. Dagley, Richard D. Unwin, Alexandra Naba, Michael J. MacCoss, Tiannan Guo, Eric W. Deutsch, Cesar Martin, Jochen M. Schwenk, Yasset Perez-Riverol

**Affiliations:** 1 European Molecular Biology Laboratory, European Bioinformatics Institute (EMBL-EBI), Wellcome Trust Genome Campus, Hinxton, Cambridge, CB10 1SD UK; 2 Department of Biochemistry and Molecular Biology, Universidad del País Vasco UPV/EHU, Bilbao, 48080 Spain; 3 Biofisika Institute (UPV/EHU, CSIC), Barrio Sarriena S/N., Leioa, Bizkaia 48940 Spain; 4 State Key Laboratory of Medical Proteomics, Beijing Proteome Research Center, National Center for Protein Sciences (Beijing), Beijing Institute of Lifeomics, Beijing, 102206 China; 5 International Academy of Phronesis Medicine (Guangdong), Guangzhou, Guangdong 510320 China; 6 Centre for Inflammation Research, Institute for Regeneration and Repair, University of Edinburgh, Edinburgh, UK; 7 Department of Proteomics and Signal Transduction, Max Planck Institute of Biochemistry, Martinsried, 82152 Germany; 8 National Institute of Standards and Technology - Charleston, Charleston, SC 29412 USA; 9 Ions.Bio GmbH, Martinsried, 82152 Germany; 10 The Walter and Eliza Hall Institute for Medical Research, Parkville, VIC 3052 Australia; 11 Department of Medical Biology, University of Melbourne, Parkville, VIC 3052 Australia; 12 Division of Cancer Sciences, School of Medical Sciences, Faculty of Biology, Medicine and Health, The University of Manchester, Manchester, UK; 13 Department of Physiology and Biophysics, University of Illinois Chicago, Chicago, USA; 14 Department of Genome Sciences, University of Washington, Seattle, WA USA; 15 State Key Laboratory of Medical Proteomics, School of Medicine, Affiliated Hangzhou First People’s Hospital, Westlake University, Zhejiang Province, Hangzhou, China; 16 Institute for Systems Biology, Seattle, WA 98109 USA; 17 SciLifeLab, Department of Protein Science, KTH Royal Institute of Technology, Solna, Sweden

## Abstract

The circulating blood proteome comprises soluble and cellular components that reflect physiological and pathological states across tissues. Advances in mass spectrometry and affinity-based proteomics have improved sensitivity and throughput, enabling the generation of public blood proteomics resources. However, comprehensive assessments of these datasets remain limited. This work reviews the cellular and molecular complexity of publicly available blood proteomics data, recent methodological developments, and the complementarity of diverse data sources across the abundance range, while outlining remaining challenges for translating blood proteomics into personalized medicine.

**Supplementary Information:**

The online version contains supplementary material available at 10.1186/s13059-026-04027-9.

## Introduction

The proteome encompasses all proteins expressed by an organism, tissue, cell, or biological fluid at a specific time and under specific conditions. While the genome provides a relatively stable blueprint of potential gene products, the proteome is highly dynamic, reflecting changes in expression during developmental, physiological conditions, disease, or the environment [1]. Proteome studies catalog and analyze proteins, their functions, interactions, and roles within biological pathways, offering insights into normal physiological processes and disease mechanisms [2]. The human proteome, despite being derived from around 20,000 protein-coding genes [3], is, however, far more complex due to protein isoforms from alternative splicing, post-translational modifications (PTMs), sub-cellular localization, and protein complex formation [4].

Blood, as a readily accessible biological fluid, serves as a primary systemic carrier of nutrients, cells, and functional molecules, making it invaluable for assessing human health and disease [5]. Its proteome offers unique insights into both physiological and pathological conditions. The analytical complexity of blood as a sample arises from its composition: the liquid phase accounts for about 55% of its volume, while the cellular phase, including erythrocytes (43%), platelets (1%), and leukocytes (1%), constitutes the rest [5]. While the cellular phase contains various cell types with unique functions, cellular proteins can enter the extracellular space through active secretion or leakage. Hence, the liquid and cellular space can be thought of as biochemically separate but interconnected compartments.

Advances in proteomic technologies have dramatically expanded the ability to identify, quantify, characterize, and catalogue human blood proteins [6]. This includes improved preparation for mass spectrometry (MS), faster and more sensitive MS instruments [7], and affinity proteomics platforms, such as Olink [8] or SomaScan [9]. All these methods have witnessed a growth in use by researchers outside the proteomics community [10]. MS-based proteomics enables the identification and quantification of thousands of proteins across studies with hundreds of samples. In contrast, non-MS platforms detect specific protein targets via the use of a defined set of affinity reagents [11]. Despite progress, the dynamic range of protein concentrations in blood, spanning 10 to 12 orders of magnitude [12, 13], still exceeds the current detection and quantification capabilities, a challenge compounded by inter-sample variability, phenotype relation, and sampling time.

Repositories integrating public proteomics data have also accelerated progress. The ProteomeXchange (PX, http://www.proteomexchange.org) consortium was established to streamline the submission and dissemination of MS data across various resources and databases, ensuring the long-term sustainability of public proteomics data [14]. Its members archive datasets and systematically reanalyze them to create catalogues of specific blood components, such as the PeptideAtlas plasma builds [13] or the quantms plasma proteome [15]. Additionally, multiple curated databases include catalogues of protein quantifications from blood proteomics datasets, including Human Protein Atlas (HPA) [16] and the Protein Abundance Across Organisms Database (PaxDb) [17]. These databases are essential resources for researchers, providing access to information on protein abundance in blood and its possible associations with disease states and phenotypes.

This review covers blood taxonomy and highlights how MS- and affinity-based proteomics approaches have driven advances in studying the circulating blood proteome. We also discuss the challenges in generating these data and the landscape of proteomics databases, including publicly available blood datasets, and the key challenges in their integration and analysis.

### A taxonomy of blood components

**Fig. 1:**
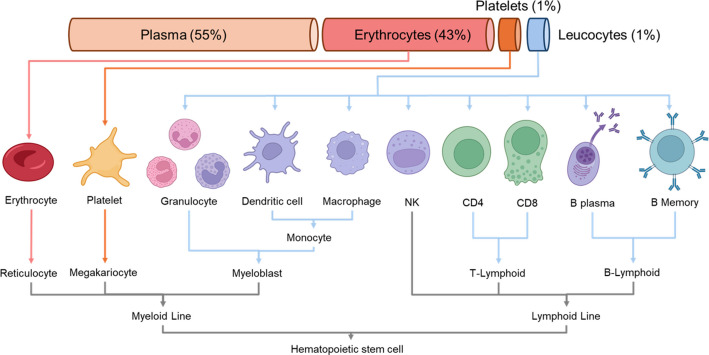
Taxonomy of blood components. The blood is composed of a liquid and cellular phase. The cellular phase of the blood consists of erythrocytes (43% of the volume), platelets (1%), and leukocytes (1%) suspended in the liquid phase (55%). Blood cells originate from hematopoietic stem cells in the bone marrow and are categorized into two lines: the myeloid line, which is the precursor of erythrocytes, platelets, granulocytes, dendritic cells, and macrophages, and the lymphoid line, which is the precursor of NK (natural killer), CD4, and CD8 T cells, B plasma cells, and memory cells

Blood plays vital roles in nutrient transport, waste removal, immune defense, and coagulation (Fig. 1). Blood circulates through a network of vessels and capillaries that connect organs and tissues, making it an invaluable resource for medical research on health and disease states [5]. Its minimal invasiveness makes blood convenient for routine health assessments, diagnosis, and biomarker investigation.

### Circulating proteome

The cell-free fraction of blood (Fig. 1) constitutes ~ 55% of blood volume, serving as a medium for transporting biomolecules and metabolic byproducts [5]. It can be prepared as plasma (anticoagulants: EDTA, heparin, citrate) or serum (after coagulation and fibrinogen removal). Although derived from the same source, plasma and serum can differ in protein concentrations [18]. Plasma is generally preferred for multi-omics due to faster preparation and reduced coagulation variability, while serum can be advantageous for certain proteomic analyses. Anticoagulant choice and other pre-analytical factors (e.g., centrifugation, processing time) strongly affect proteome detection, especially for low-abundance proteins and extracellular vesicle signal [19]. We use the term circulating blood proteome to combine these preparatory aspects, distinct from the cellular blood proteome. The circulating proteome includes proteins actively secreted or shed, as well as those passively released from tissue and cellular damage [6]. The secretome comprises proteins secreted via classical or non-classical pathways important for signaling, immunity, and homeostasis [20]. Tissue-leakage proteins enter the circulation passively and serve as indicators of organ injury (e.g., cardiac troponins, liver enzymes) commonly used in clinical diagnostics [21, 22]. In addition to freely soluble proteins, the circulating proteome also contains extracellular vesicle (EV)–associated proteins, which reflect the intracellular state and tissue origin of their parent cells and can provide complementary information to bulk plasma proteomics [6].

Although the circulating proteome is estimated to comprise 6,000 to 10,000 proteins (excluding proteoforms), this number reflects the collective output of multiple proteomic approaches and datasets rather than what can be detected in every sample [23]. Upper bound estimates derive from bioinformatic predictions of secretion-associated features (e.g., SignalP or SecretomeP) [24]. However, the actual number of actively secreted and soluble proteins is around 2,500 [20], once factors like intracellular contamination or extracellular matrix (ECM) incorporation are considered [25]. Although large-scale studies may report thousands of detectable proteins, only a subset can be quantified with consistency and confidence; estimates are often inflated by artifacts or contamination, underscoring the need for stringent QC and cautious data interpretation. A major challenge is detecting low-abundance proteins masked by the exceptionally wide dynamic range of plasma proteins [13]. This imbalance is driven by a few highly abundant species, including albumin (~ 40 g/L; 50–60% of the total protein mass in the liquid phase) and immunoglobulins (~ 35–40%) [26]. Beyond technical limitations, biological variability complicates analysis: protein levels fluctuate diurnally and with food intake, physical activity, and stress [27].

### The cellular blood proteome

The cellular proteome comprises proteins from blood cell types with specialized functions: oxygen transport (erythrocytes), coagulation (platelets), and immune defense (leukocytes) (Fig. 1). Characterizing the cellular proteome is critical for understanding normal physiological and immune functions and shifts in its profile can serve as indicators of infection or immune-related disorders. Erythrocytes constitute ~ 43% of blood volume (varying by sex, age, and health status) and transport oxygen via hemoglobin [28]. Platelets, although < 1% of blood volume, play key roles in hemostasis and wound healing [29]. Leukocytes, also < 1% of blood, include T and B cells, natural killer (NK) cells, granulocytes, monocytes, dendritic cells, and macrophages, with diverse subtypes and activation states each having distinct proteomes and functions [30]. Neutrophils alone account for 60–70% of leukocytes, highlighting their predominance within this heterogeneous group [31].

Analyzing the proteomes of specific blood cell types offers critical insights into physiology and disease. Erythrocyte alterations link to hemoglobinopathies, anemia, cardiovascular disease, and diabetes [32]. Platelet proteomics informs hemostasis, thrombosis, and immune regulation [33], and leukocyte dynamics provide biomarkers of infection, inflammation, autoimmunity, and cancer [30, 34, 35]. MS-based plasma/serum profiling often detects unintended cellular proteins, complicating separation of true circulating signals from handling artifacts; even carefully prepared samples contain proteins from normal turnover that may be genuine biomarkers or confounders [29]. Geyer et al. [25] established quality marker panels for erythrocytes and platelets, and proteins altered in coagulation. Their analysis of > 200 published studies found that > 50% reported at least one potential cellular quality marker as a biomarker candidate. Gao et al. [36] and Korff et al. [37] showed that platelet and erythrocyte proteins can be introduced by sample processing methods, and that leukocytes are another detectable source with modern MS-based approaches.

## Technological approaches to blood proteomics

### Mass spectrometry

Modern MS analytical methods can identify and quantify proteins with high precision up to 7,000 proteins in biological fluids [38] and > 10,000 in some cell types [39]. To address the complexity and dynamic range of the circulating proteome, sample preparation strategies have been developed to reduce sample complexity, facilitating deeper proteome coverage [6]. One widely used approach is depletion of high-abundance proteins, such as albumin, immunoglobulins, and fibrinogen, which together account for > 99% of total liquid phase protein mass [40]. An emerging complementary enrichment strategy uses nanoparticles with tunable surface chemistries to form protein coronas that selectively adsorb proteins from liquid samples, enabling enrichment of low-abundance species without immunodepletion or extensive fractionation [41]. These approaches capture thousands of proteins based on physicochemical properties and include platforms such as Seer’s Proteograph, Mag-Net, ENRICH/ENrichPlus [41]. However, bead-based enrichment is highly sensitive to cellular contaminants, which can preferentially bind to beads and distort protein capture; even minimal contamination can introduce thousands of additional proteins and obscure low-abundance biomarkers, with platelet proteins being particularly problematic [37, 42]. Further non-affinity methods like perchlorate precipitation, can also increase the number of identified plasma proteins. Beyond circulating proteome analysis, MS is widely used to profile specific blood cell types, typically following high-purity isolation by methods such as FACS [43], while recent advances in nanoLC-MS and sample processing have enabled emerging applications in single-cell proteomics [44].

MS can also detect PTMs, splice isoforms, and proteoforms, offering biologically and clinically relevant insights into the blood proteome. Phosphoproteomics has mapped dynamic signaling networks in T lymphocytes, while large-scale resources such as PTMD document thousands of disease-associated PTMs across many human proteins [45]. MS has identified PTM- and isoform-specific protein variants in plasma and blood cancers, demonstrating access to molecular states largely invisible to affinity-based methods and underscoring its unique value for biomarker discovery and functional proteome analysis [46]. MS-based glycoproteomics further allows site-specific characterization of protein glycosylation, revealing meta-heterogeneity across glycosylation sites [47].

### Affinity-based proteomics

Affinity-based approaches use antibodies or aptamers for sensitive and specific protein detection, particularly in fluids. A prominent example is the Proximity Extension Assay (PEA), as implemented by the Olink platform, which combines dual antibody recognition with oligonucleotide-tagged probes. SOMAmer-based assays follow a similar principle using aptamers instead of antibodies, while newer technologies like NULISA enhance sensitivity through nucleic acid tagging [48]. Affinity proteomics offers targeted scalability and high-throughput capacity, making it ideal for profiling larger cohorts and clinical studies. To date, these techniques have been applied to more than 50,000 samples in a UK Biobank (UKB) study [31], with an upcoming project to expand this to more than 600,000 samples using affinity-based proteomics. While some of these current assays target around 10,000 proteins, in practice, only a subset are reliably detected and quantified [38].

Affinity-based approaches are limited to predefining the targets, as they require the generation and testing of specific binding reagents. This makes them particularly unsuitable for determining unknown proteins, isoforms, or PTMs. Affinity reagents may also be susceptible to off-target binding or reduced specificity in a given sample, especially in complex mixtures. Individual validation of each individual affinity ‘pair’ scale is needed to ensure data accuracy and reliability, and ensure more efficient and reliable translation of candidate biomarkers into more usable, smaller-scale clinical assays [49]. Indeed, comparative studies between platforms such as Olink and SomaScan, or validation against singleplex ELISA, which are highly optimized on one selected target, have shown that only around half of the measured proteins show strong concordance, highlighting the need for orthogonal validation in biomarker discovery [50].

### Comparative overview of MS and affinity proteomics

Comparing proteomics techniques is essential for assessing the differences in platform-specific performance and for understanding how the reported biology shapes biomarker discovery. Large-scale studies comparing affinity-based platforms with MS approaches have shown limited overlap in protein detection, quantification, and trait associations [38, 51]. Even for the same proteins, quantitative values often diverge, indicating that discrepancies extend beyond coverage to measurement accuracy. These contrasts are especially evident in differential-abundance analyses, where both MS and affinity assays detect robust disease- or trait-associated signatures but frequently identify largely non-overlapping sets of proteins [38]. In some cases, affinity platforms report more differential proteins, often involving lower-abundance targets, while MS tends to identify fewer changes but with higher reproducibility and stronger replication across studies [52]. When analyses are restricted to proteins reliably quantified by both approaches, however, the agreement in effect direction and magnitude increases markedly [52], indicating that platform-specific sensitivities, rather than biological contradiction, drive most discrepancies. Together, these findings show that each technology captures distinct proteomic features based on detection principles, assay specificity, and analytical precision, limiting their interchangeability [53]. Thus, platform choice should depend on the biological question, as no single method provides comprehensive or universally applicable coverage [54]. Additional file 1: Table S1 summarizes key comparisons between platforms and outlines their relative strengths and limitations as understood at the time of writing.

## The blood proteome in public data

**Fig. 2:**
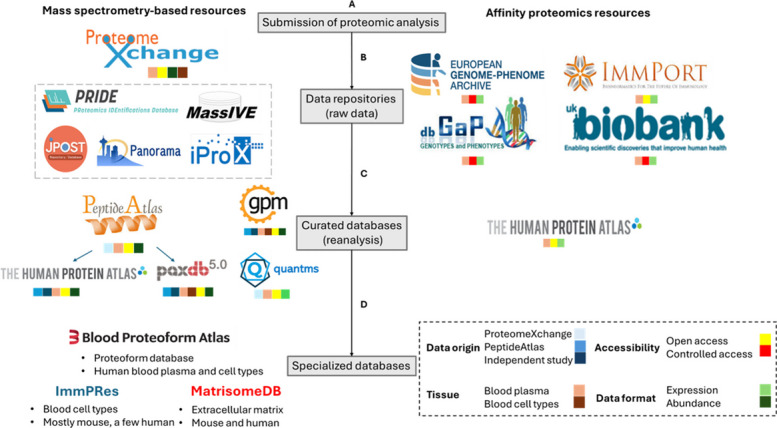
Schematic overview of major proteomics resources. **A** Submission of proteomics analyses. Researchers deposit their raw proteomics datasets into online repositories to ensure long-term storage and accessibility. **B** Data archives (raw data). While repositories primarily serve to store and provide access to raw datasets, they do not typically reanalyze or harmonize the data. For MS-based studies, most repositories are open access, with the ProteomeXchange consortium serving as the central hub. ProteomeXchange includes several partner repositories (e.g., PRIDE, MassIVE, jPOST, Panorama) and hosts data from both the circulating proteome and blood cell types. For affinity proteomics, there is no unified repository. Instead, datasets are stored in resources such as the European Genome-phenome Archive (EGA), dbGaP, and the UK Biobank, all of which require controlled access, or ImmPort, which is open access. Since antibody-based methods do not reliably capture cellular proteomes, these datasets are largely restricted to the circulating proteome. **C** Curated databases (reanalysis). In contrast to repositories, databases analyze, reprocess, and integrate proteomics data to make them more interpretable and comparable across studies. In MS-based proteomics, examples include PeptideAtlas, Human Protein Atlas, PaxDb, GPMDB, and quantms. These differ in data origin (direct from repositories or from other curated databases), analyzed tissue (plasma, cell types, or both), and data format (protein abundance or expression). Notably, Human Protein Atlas and PaxDb both rely heavily on PeptideAtlas, which itself reanalyzes data from ProteomeXchange. For affinity proteomics, curated databases are rare; the Human Protein Atlas is the main resource that integrates antibody-based protein data. **D** Specialized databases. Some resources focus on specific biological niches. Examples include the Blood Proteoform Atlas (circulating proteoforms), ImmPres (immune cell proteomes), and MatrisomeDB (extracellular matrix proteins). These specialized resources provide higher-resolution insights for targeted areas of biology and medicine. The legend also includes a symbol-coded annotation system, indicating for each resource its data origin (independent vs. repository-based), analyzed tissue (plasma, cell types, or both), data type (expression or abundance), and accessibility (open vs. controlled)

Moving forward, a crucial component of proteome research is publicly available datasets and resources providing raw mass spectra, peptide identifications, PTMs, and protein expression profiles. Proteomics research relies on data repositories and databases for accessible, reusable, and properly curated datasets, and blood proteome research is not an exception [55]. Proteomics data resources can be broadly categorized into data repositories, storing raw and original results; protein databases, which provide standardized, reanalyzed data from archives; and knowledgebase resources, which integrate proteomics data with broader biological information such as protein function, structure, and genomic context (Fig. 2). The typical data flow among these resources begins with researchers depositing their datasets, including raw data, metadata, and experimental descriptions, into public archives (ProteomeXchange [14], PRIDE [56], or MassIVE [57]). Protein expression databases then reanalyze and standardize these data using uniform workflows, making expression information accessible (PeptideAtlas [58] or HPA [16]). Finally, broader knowledge base resources such as UniProt, Ensembl, and Reactome, integrate this information into a comprehensive biological context.

### ProteomeXchange

To standardize the sharing of MS-based proteomics data, the ProteomeXchange Consortium (https://www.proteomexchange.org/) was established as a global framework for the dissemination of MS datasets. As of 2025, the ProteomeXchange consortium stores more than 44,000 publicly released MS-based datasets (and over 20,000 datasets not yet publicly released), including 20,600 human studies. Notably, 826 human blood liquid phase datasets have been uploaded to ProteomeXchange, with up to 75% originating from the PRIDE repository (Table 1). While the blood plasma and serum proteomes are among the most extensively studied sample types, cellular components such as platelets (125 datasets) and NK cells (19 datasets) remain underrepresented, limiting the ability to build comprehensive proteomes for individual cell types.

**Table 1:** Datasets available by blood components and cell types, in ProteomeXchange, and corresponding partners. The absence of a dataset does not indicate that the archive lacks datasets of that type (-); rather, it suggests that they cannot be easily filtered or searched

Database	Liquid phase	Erythrocytes	Platelet	Monocytes	Dendritic cells	NK	Neutrophil	T cell	B cell
PX	826	57	125	82	32	19	96	26	96
PRIDE	624	51	103	76	29	18	84	22	83
MassIVE	41	4	5	2	2	1	3	1	5
Panorama	19	1	-	-	-	-	-	-	-
iProX	92	1	14	-	2	-	8	3	7
jPOST	30	-	1	1	-	-	1	-	2

Among the most comprehensive MS-based studies of the circulating proteome to date, recent work has reported over 7,000 unique proteins across 40 samples, with up to 4,500 proteins per individual [38]. Regarding blood cell-type proteomics, MS remains the primary technology, with recent studies identifying a collective of > 10,000 proteins, depending on the specific cell type and analytical depth. However, like many of the current MS-based studies of plasma or serum, the sample sizes in these cell-specific datasets remain limited. Additional file 2: Table S2 highlights notable datasets using MS or affinity-based technology based on the number of reported proteins, with a focus on liquid samples and specific blood cell types.

### PeptideAtlas

PeptideAtlas (https://peptideatlas.org/) was initiated in 2004 and has become one of the leading resources from the Human Proteome Organization for developing the Human Proteome Project. The 2025–08 Plasma PeptideAtlas build includes 158 datasets and 4,583 canonical proteins, excluding isoforms and immunoglobulins. PeptideAtlas has stringent false-positive filtering across the entire build and quantifies proteins using both raw counts and a normalized metric, with this study relying on the latter (a comparison of both methods is available in Additional file 3: Fig. S1). Additional file 4: Data S1 provides additional information, including links to the original datasets and a GitHub repository containing the data used in this study.

### Human protein atlas

The Human Protein Atlas (HPA, https://www.proteinatlas.org/) was launched in 2003 to map all human proteins in tissues and cells using antibody-based methods. The HPA offers open access to data about eight different sample types, including single cells, blood, and cell lines, among others. In 2025, HPA offers a blood proteome resource, including PEA data from a longitudinal wellness study, including data for 2,910 proteins [59]. The portal also presents quantitative immunoassay data of 308 proteins secreted into blood, as well as MS data currently based on the 2025–08 build of PeptideAtlas and presented in a gene-centric view of 4,286 proteins. HPA also provides data on circulating proteins across 59 diseases and 6,121 patients, in a pan-disease study currently covering 1,463 proteins by PEA and 146 proteins by targeted MS (Additional file 4: Data S1).

### PaxDb

PaxDb (https://pax-db.org/) is a comprehensive resource launched in 2012 that aggregates and normalizes protein abundance data across multiple organisms and tissues and resources, focusing on creating a consensus view on normal/healthy proteomes. The most recent release of PaxDb (version 6.0, 2023) accounts for 26 plasma proteome builds, including older PeptideAtlas versions (up to 2021), and several independent studies (PXD010899 [60], PXD004242 [61]). PaxDb also integrates all plasma builds in one, with a total number of 7,454 quantified proteins. A specific comparison of the plasma-centric sources used in the PaxDb is available in Additional file 5: Fig. S2. PaxDb also contains proteomic data about different blood cells, such as monocytes (5,265 quantified proteins), B cells (9,392), NK cells (8,931), CD4 (7,717), or CD8 (11,107), most of them collected from a single study (PXD000561) [62] (Additional file 4: Data S1).

### GPMDB

The GPMDB (https://www.thegpm.org) was launched to facilitate the analysis and interpretation of MS data in proteomics research. It was developed as an open-access platform to support protein identification and annotation, with a focus on the integration of peptide and protein data from tandem MS (MS/MS) experiments. GPMDB provides several specialized builds, including the complete human proteome categorized by chromosome, as well as detailed datasets on acetylation and phosphorylation sites. Additionally, GPMDB features a build that classifies human proteins by tissue type, covering plasma (5,154 proteins), serum (7,945), erythrocytes (617), platelets (7,281), and more (Additional file 4: Data S1).

### quantms

The quantms is a new open-source, cloud-based bioinformatics pipeline for massively parallel reanalysis of public proteomics data. The reanalyzed datasets are made available through quantms.org (https://quantms.org/datasets), serving as a hub for well-annotated in SDRF [63] and reanalyzed proteomics data. Data is reanalyzed using the quantms workflow, supporting DDA label-free (DDA-LFQ), DDA isobaric (e.g., TMT), and DIA label-free. Proteins are filtered at 1% FDR with ≥ 2 unique peptides, and their intensities are summarized into iBAQ values using SDRF for normalization and batch correction [64]. quantms has reanalyzed 114 public ProteomeXchange datasets, including 19 large-scale plasma experiments, totaling 1,296 plasma samples and 2,799 quantified proteins.

### The blood proteoform atlas

The Blood Proteoform Atlas (BPA, https://blood-proteoform-atlas.org/) [65] represents the first systematic mapping of intact proteoforms across human hematopoietic cells and plasma, offering a significant advance in proteome resolution. Unlike traditional protein-level measurements, which often conflate multiple biological variants into a single entry, the BPA captures distinct proteoforms, the molecular forms that proteins take due to PTMs, alternative splicing, and other processing events. By 2025, the BPA reports 29,620 nonredundant proteoforms mapped to 1,690 unique proteins, identified across 21 cell types and plasma. On average, each gene gives rise to 17.5 proteoforms, highlighting the considerable molecular diversity encoded beyond the transcriptome. Importantly, proteoforms demonstrate greater cell-type specificity than proteins: while most proteins are detected in multiple cell types (81%), a majority of proteoforms (58%) are found in only one. This specificity suggests that proteoforms may serve as more accurate markers of cellular phenotype and state than protein-level measurements alone.

### Other MS databases

MatrisomeDB (https://matrisomedb.org/) [66] is a curated resource focused on human and mouse proteomic datasets characterizing the ECM, or matrisome, of tissues. The ECM is a network of structural proteins, such as collagens and fibronectin, and regulatory proteins modulating core ECM protein structures and functions [67]. While typically associated with solid tissues, many ECM proteins are part of the liquid phase of the blood and assemble as a solid matrix upon clotting [68]. MatrisomeDB supports blood proteome research by cataloging matrisome proteins identified in plasma, aiding biomarker discovery in contexts like fibrosis and cancer. ImmPRes (http://immpres.co.uk/) [69] is a resource for immune cell proteomics, mainly in mice but with limited human data, providing curated profiles to study gene regulation, signaling, differentiation, and immune-specific biomarkers.

### Non-MS repositories and databases

The largest affinity-proteomics dataset generated to date comes from the UKB [70], where the Pharma Proteomics Project has assayed plasma samples from 54,000 participants using the Olink platform, quantifying approximately 3,000 circulating proteins [71]. Unlike resources such as PeptideAtlas or the HPA, which aggregate and reanalyze raw MS datasets, UKB produces its own proteomic data directly from its cohort. These measurements are linked with extensive genomic, imaging, lifestyle, and health-record information, enabling large-scale protein quantitative trait locus mapping, biomarker discovery, and drug-target validation. Access to these data is controlled and requires an approved application to UKB.

Beyond the UKB, a limited number of repositories currently host affinity-proteomics data. Examples include PRIDE Affinity Archive (https://www.ebi.ac.uk/pride/archive/affinity-proteomics), the European Genome-phenome Archive (EGA; https://ega-archive.org/) [72] or Immunology Database and Analysis Portal (ImmPort; https://www.immport.org/home) [73]. At present, PRIDE Affinity Archive contains 9 Olink and 6 Somascan datasets, EGA contains 7 Olink and 6 Somascan datasets, while ImmPort hosts 19 Olink and 8 Somascan datasets. Another resource relevant to proteomics is the Database of Genotypes and Phenotypes (dbGaP; https://www.ncbi.nlm.nih.gov/gap/), which contains 3 Olink datasets. The increasing impact of affinity-proteomics platforms is also seen in the literature: a PubMed search for “SomaScan” results in 423 publications, while a search for “Olink” shows 1,085. Despite this expanding body of work, no dedicated public archive currently exists to systematically store and share affinity-based proteomics data.

## Charting the blood proteome using public databases

Herein, we systematically compare multiple public data sources and independent ProteomeXchange datasets, highlighting their complementary strengths, inherent limitations, and the persistent challenges in constructing a unified, accurate map of the human blood proteome. Methods for processing the original data are detailed in Additional file 6: Data S2.

### Circulating proteome: PeptideAtlas, HPA, PaxDb, GPMDB, quantms

**Fig. 3:**
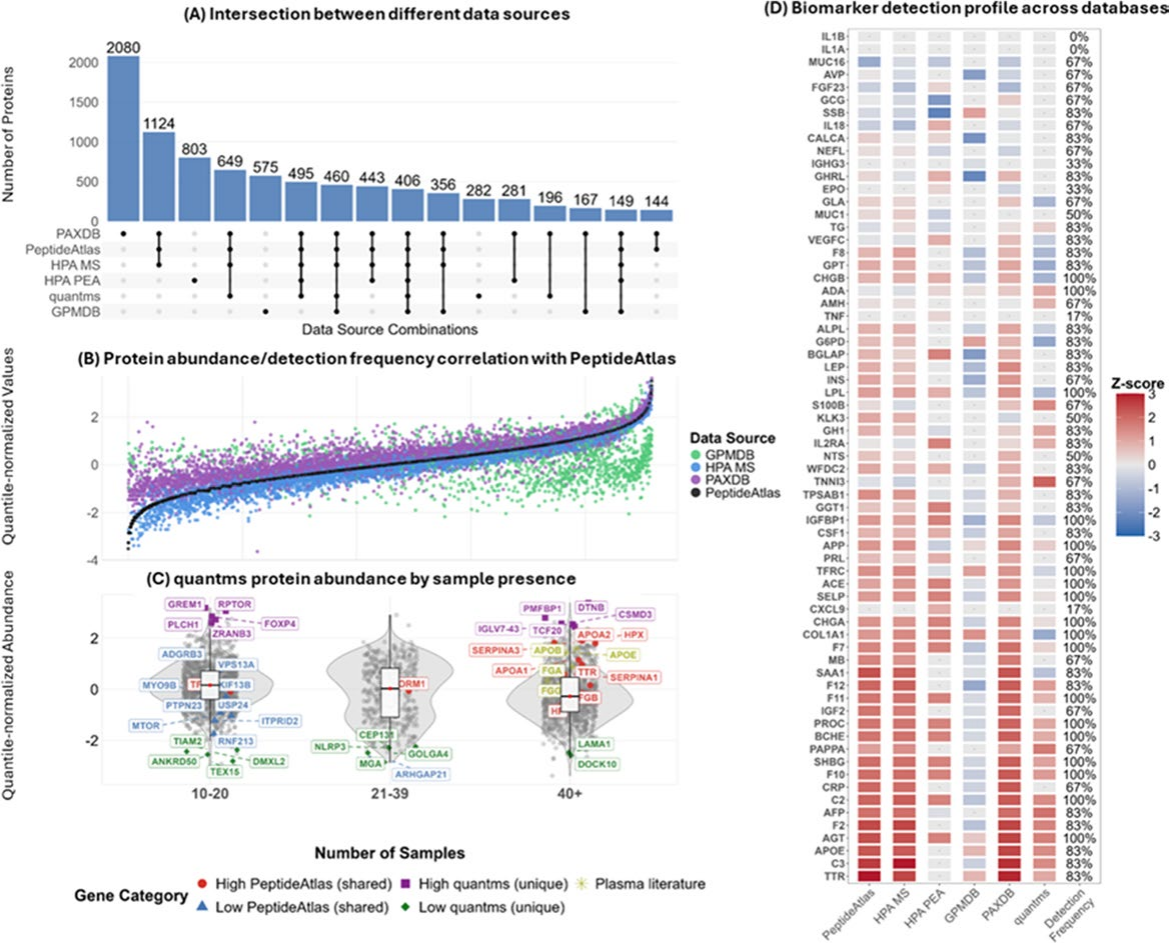
Comparative analysis of plasma proteomic databases. **A** Overlap of identified proteins across databases. Combinations identifying fewer than 140 proteins were excluded for clarity. **B** Comparison across abundance databases (PeptideAtlas, PaxDb, HPA-MS, and GPMDB). Proteins are ordered by increasing abundance in PeptideAtlas, which serves as the reference dataset. **C** quantms values grouped by sample abundance. Proteins were categorized into three groups based on the number of samples in which they were identified. Highlighted protein sets include proteins from the circulating proteome known from the literature (yellow), high-abundance proteins from PeptideAtlas shared with quantms (red), low-abundance proteins from PeptideAtlas shared with quantms (blue), high-abundance proteins unique to quantms (purple), and low-abundance proteins unique to quantms (green). **D** Z-score intensities and abundance of 65 known blood liquid phase biomarkers (from MarkerDB 2.0), sorted by their relative concentration in PeptideAtlas, showing their detection across PeptideAtlas, HPA MS, PaxDb, GPMDB, HPA PEA, and quantms datasets. Z-scores are shown by color intensity. Grey dots indicate non-detections

We analyzed plasma protein abundance and expression across multiple resources, including PeptideAtlas, GPMDB, PaxDb, quantms, and the HPA, encompassing MS and PEA (Fig. 3). PaxDb reported the highest number of proteins (7,235), followed by PeptideAtlas (4,584), gene-centric HPA MS (4,285), quantms (2,799), and GPMDB (2,266), completing the MS-based dataset group. The HPA projects used the antibody-based PEA platform to target 2,871 proteins. Integration of all six datasets yielded 9,237 unique proteins. This count reflects a simple aggregation across studies, without reanalysis or global FDR correction, and should be viewed as a cumulative resource rather than a rigorously validated circulating proteome. Of these proteins 60% are reported in more than two sources, while 40% appear in only one. Only 406 proteins are shared across all six datasets (Fig. 3A, Additional file 7: Data S3), indicating that no single database fully encompasses all proteins, and multiple sources are required for comprehensive plasma coverage.

Besides identification, these databases offer quantitative data for each protein. In PeptideAtlas, PaxDb, HPA-MS, and GPMDB, normalized spectral counts or the number of protein observations serve as proxy measures of protein abundance [74]. Conversely, quantms provides normalized intensity-based expression values for each protein, independent of the number of observations (hereafter referred to as baseline expression). There is a noticeable concordance in classifying highly abundant proteins across databases (Fig. 3B). While any of these sources can be used to study protein abundance in plasma, quantms could be used to study the baseline expression of any given protein and its variability across plasma samples (Fig. 3, Additional file 8: Data S4). Figure 3C shows that well-known, high-abundance plasma proteins like ApoB and FGG (represented in yellow) are predominantly quantified in a high number of samples in quantms (≥ 40 samples). As expected, proteins with low abundance in PeptideAtlas (in blue) largely fall into the low-abundance group in quantms, implying that these low-abundance proteins present a consistent detection challenge across both resources. Figure 3C also shows proteins unique to quantms (green), spanning low- and high-abundance groups. A similar but reduced comparison of serum databases is analyzed in Additional file 9: Fig. 3S.

To explore the relationship between protein abundance and biomarker detection, we analyzed the distribution of 65 known biomarkers curated from MarkerDB 2.0 (Additional file 10: Data S5 for details on filtering and the list) [75]. Figure 3D shows the Z-score quantification values (abundance or expression) of these biomarkers. PeptideAtlas, HPA MS, and PaxDb identified the highest number of biomarkers (61 out of 65) reflecting overlapping protein coverage, followed by GPMDB, HPA PEA, and quantms. PeptideAtlas-based databases yield fewer identifications at low concentrations compared to GPMDB and HPA PEA. PEA, in particular, omits many high-abundance proteins by design, as they are often less informative for expanding the horizon from a more disease-specific analysis [8]. On the other hand, quantms shows a moderate similarity to PeptideAtlas-based databases but misses several low-abundance biomarkers. Only 17 of 65 blood biomarkers were detected across all six databases, and only 9 biomarkers consistently showed the same relative abundance trend, either above or below average Z-score, in all the databases where they were detected.

### Cellular blood proteome databases

**Fig. 4:**
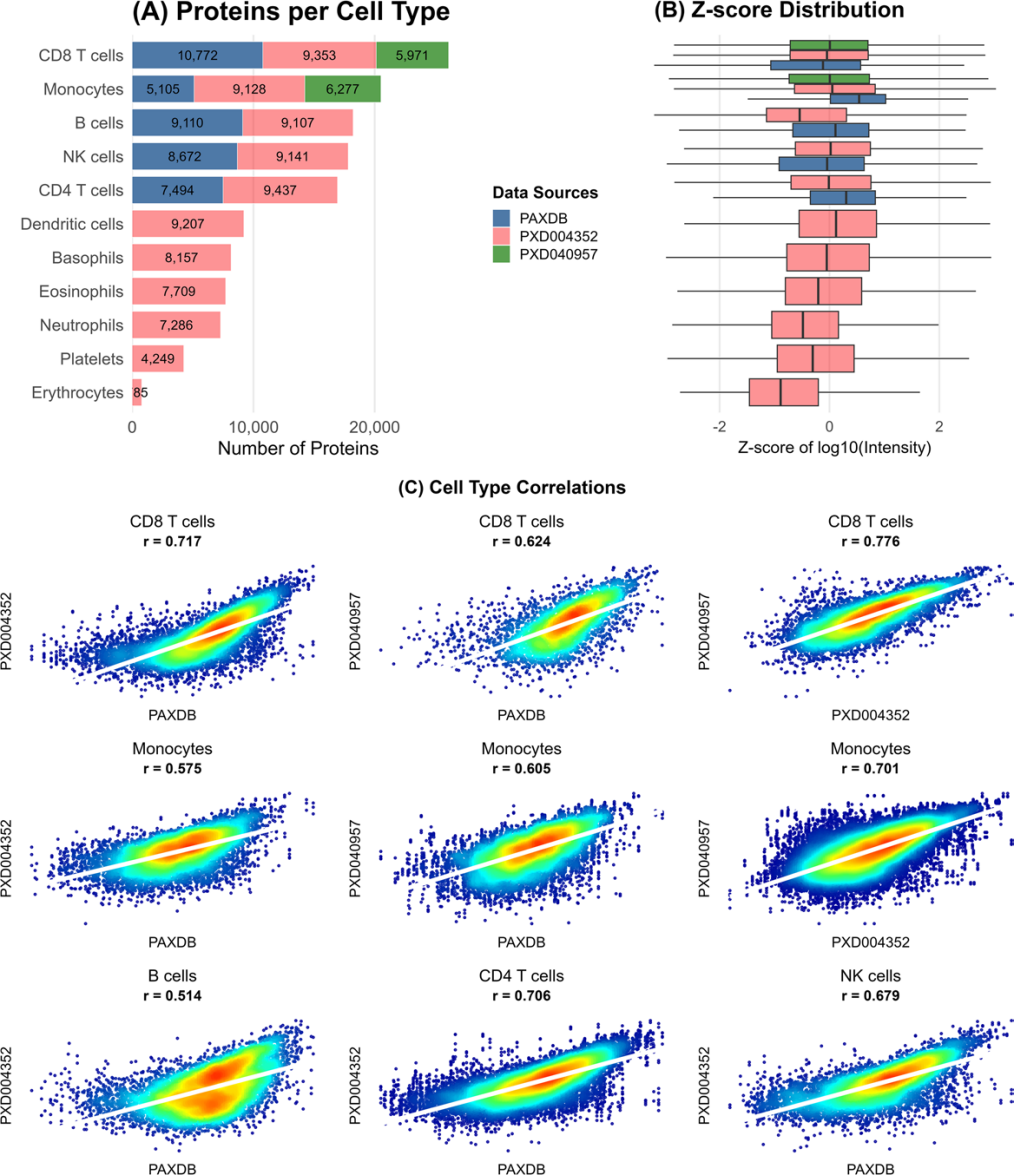
Overview of data across cellular blood proteomes. **A** Number of proteins reported per cell type in each dataset or database, including PaxDb and ProteomeXchange datasets (PXD004352, PXD040957). **B** Distribution of protein expression values across cell types for each resource. **C** Pairwise correlation of protein concentration changes across databases, using a binary comparison system

The global landscape of the blood cell proteome has mainly been studied using MS-based technologies and targeted affinity-based studies that investigate specific cell type populations. Figure 4 presents the proteins reported across a cell-type-specific resource (PaxDb), as well as relevant original datasets deposited in ProteomeXchange (PXD004352 [76], PXD040957 [77], Additional file 4: Data S1 and Additional file 11: Data S6). The proteomes of all subtypes have been combined and averaged, allowing for a comparative analysis across the datasets. The procedures used to process, harmonize, and integrate data across databases are detailed in Additional file 6: Data S2.

Most studies reported 7,000–9,000 proteins per cell type, except erythrocytes and platelets (Fig. 4A). PXD004352 stands out by reporting a higher number of proteins, largely due to its analysis of multiple subtypes, although it should be noted that this study relied on a permissive matching strategy without FDR control, which may have inflated the reported counts. CD8 +, Monocytes, CD4 +, B cells, and NK exhibit the highest protein counts, while erythrocytes and platelets have the fewest. The proteomes were also combined and compared to evaluate overlap in protein reports (data of the combined databases per cell type are available in Additional file 12: Table S3; overlaps in Additional file 13: Fig. S4). Combining multiple resources raised the total to approximately 6,000–13,000 proteins. No global FDR correction was applied in this integration step, so the aggregated list should be interpreted as a cumulative resource rather than a rigorously validated proteome [78]. Notably, 40–50% of each cell type’s combined proteome was reported in only a single dataset. Strikingly, for platelets and erythrocytes, only 17% and 1% of their combined proteomes, respectively, were shared between GPMDB and PXD004352 (Additional file 14: Data S7). Across cell types and resources, expression distributions were broadly consistent (Fig. 4B). Using PXD004352 as a baseline (covering all cell types), PXD040957 showed a similar distribution, while PaxDb displayed greater variability. Compared with plasma and serum, cell types showed a more cohesive proteomic profile, though broader data may reveal more complexity. Protein concentration changes showed moderate correlations (0.5–0.7) for most database pairs, highest in CD8 + cells and lowest in B cells (Fig. 4C). We also evaluated the BPA database, which profiles blood proteomes at the proteoform level. Its coverage is limited (400–700 proteins per cell type, ~ 4–7% of the total proteome; Additional file 15: Table S4), but the overlap with our data is high (70–97%), supporting the reliability of its identifications. The limited depth likely reflects current constraints of top-down proteomics. Thus, BPA provides valuable, high-confidence insights into proteoform diversity but serves best as a complement to comprehensive bottom-up datasets.

## Conclusions and future challenges

The circulating blood proteome holds immense promise for diagnostic and prognostic biomarker discovery and personalized medicine. Despite growing popularity, characterizing the blood proteome faces major challenges due to its broad dynamic range and substantial biological variability between soluble and cellular components.

Within the proteomics community, MS remains the most established and widely implemented technology for accessing both circulating and cellular blood proteomes, supported by a mature infrastructure of platforms, databases, and repositories. With recent advances, including improved enrichment strategies and next-generation instruments, MS-based studies can now identify over 7,000 proteins from a single plasma cohort [38] as well as investigate other sample or tissue types, post-translational modifications, and novel protein discovery. Despite this progress, high sample throughput MS analysis has been limited by the cost, time, and technical expertise required. This bottleneck is gradually easing; recent plasma MS studies now encompass hundreds of individuals, reaching cohorts of 800 or even 50,000 samples [79–81]. Although large population-scale studies remain to be published as of today to match the scales achieved by affinity platforms, recent advances demonstrate a clear trajectory toward larger, more statistically powered MS-based studies.

The sample throughput challenges are even more pronounced when MS is applied to blood cell types. Most major blood cell populations are currently supported by only dozens of publicly available MS studies and datasets. The lack of blood cellular proteomics datasets is the result of technical and biological constraints: isolating pure cell populations is labor-intensive, yields can be limiting, and the diversity of immune and hematopoietic subtypes complicates standardization [43]. Additionally, the insufficient standardization, metadata in public repositories or the wrong annotation of datasets could impact the findability of these datasets [63, 82]. Many more datasets are likely deposited in public repositories, but poor labelling, incomplete annotation, or inconsistent use of controlled vocabularies make them difficult to discover or reuse. Addressing this issue requires improvements not only from researchers when submitting data, but also from repositories, which must provide clearer standards like SDRF [63], mandatory metadata fields, and better tools for dataset curation and accessibility.

Despite these challenges, the current scarcity of cellular blood proteomics data represents a major opportunity for MS-based research. Expanding MS-based studies to cover the full range of immune and hematopoietic cell types is a natural next step and takes advantage of MS’s unique capabilities, unlike affinity-based technologies, which are largely restricted to liquid samples. More comprehensive and better-annotated cellular reference maps generated through such efforts would also help distinguish proteins genuinely present in circulation from those introduced through sample handling, cell activation, or low-level contamination, an essential step toward defining a biologically accurate plasma proteome [37]. While spectral libraries and reference proteomic datasets have been developed for key human immune cell types to support targeted MS workflows (e.g., spectral assay libraries for primary T, B, and NK cells) [83], dedicated and broadly curated databases focused specifically on blood cell proteomes are still lacking. Existing cellular proteomics databases often rely on only a single or a very limited number of datasets, limiting their generalizability and utility. Building comprehensive, cell-type–resolved resources would therefore fill a critical infrastructural gap and enable more effective integration, comparison, and reuse of emerging cellular proteomics data.

Similar to blood cell types, EVs pose substantial analytical challenges for MS due to their low abundance and co-isolation with highly abundant plasma components, resulting in limited proteome coverage and standardization [84]. Although robust MS-based EV datasets are scarce, and therefore not covered in detail in this review, emerging community efforts and resources, such as cEV-map, aim to systematically characterize circulating EVs and highlight their potential as a complementary dimension of blood proteomics [85].

Affinity-based proteomic platforms, most prominently Olink and SomaScan, have already transformed plasma proteomics by enabling, for example, genome-wide association analyses to link genetic variants to plasma protein abundances at a population scale [11]. Their latest assay generations can target up to ~ 10,000 proteins in plasma [38] and have been applied to cohorts exceeding 38,000 individuals [31]. Despite this impressive throughput, these platforms face limitations. They are limited to the analysis of liquid samples, they rely on predefined target panels, lack specific assays to detect post-translational modifications, are not made to discover novel proteins, and most often still provide relative rather than absolute quantitative values. Similar to MS, the lack of global standards complicates biological interpretations and clinical implementation. Additionally, the assays’ specificity depends mostly on the performance of the affinity reagent, and systematic large-scale, systematic validations are still ongoing to ensure that each reagent reports the abundance of the intended target rather than possible off-target binding [50, 53]. The utility of affinity-based data for an even broader community is further constrained by the current immaturity of supporting repositories: emerging repositories such as the PRIDE Affinity Archive, EGA, and ImmPort contain only a limited number of entries, while curated resources, including affinity-focused efforts within the HPA, often rely on single studies. Expanding and strengthening these repositories and databases will be essential to support data sharing, standardization, large-scale benchmarking, and integration with other proteomics modalities, ultimately enhancing the reliability, reproducibility, and impact of affinity-based research.

Technological advances in MS and affinity-based proteomics have dramatically expanded our ability to explore the circulating proteome in humans. However, with this increased depth comes a new challenge: while we can now detect thousands of proteins in individual studies, integrating these results across datasets reveals substantial inconsistencies. Individual studies using either MS or affinity-based approaches report 7,000 to nearly 10,000 proteins in a single dataset, respectively [38], and by combining multiple datasets and databases, in this study, we reached over 9,000 proteins. In contrast, the largest high-confidence, curated database (PeptideAtlas) currently lists only around 4,600 proteins due to stringent quality controls [79]. This discrepancy illustrates that, although we can now achieve remarkable depth in proteome coverage, variability across studies remains a major obstacle, and the number of proteins detected per sample, and in all samples should be reported instead of the total number across the study [6, 25]. Factors such as differences in pre-analytical sample handling, experimental design, instrumentation, quantification strategies, data analysis pipelines, and the inherent broad dynamic range of the circulating proteome all contribute to this variability [86], making it difficult to establish a coherent and biologically meaningful reference [87]. Addressing these challenges will require careful normalization, standardization, and cross-study integration.

Building on these insights, future progress will increasingly rely on high-quality, technology-agnostic reference samples and standardized frameworks. These resources can provide common benchmarks across studies and platforms, improving dataset integration and reducing discrepancies from sample handling and instrumentation [87]. Building an AI-ready ecosystem [88], with standardized formats [63], shared pipelines, and benchmarking datasets, will further enhance integration across platforms. Applied to large cohorts, single cells, and the blood liquid phase, AI-driven analyses will be a navigator to uncover complex proteomic signatures and translate discoveries into clinical applications. The human blood proteome is becoming increasingly charted thanks to public datasets, but executing its full potential will only be realized as technologies improve and the surrounding ecosystem, databases, repositories, reference standards, and AI tools evolve alongside them. Together, these advances will transform public proteomic data into reliable, actionable insights for research and clinical applications.

## Supplementary Information

Additional file 1: Table S1. Comparative overview of proteomic techniques used in blood analysis at the time of writing. This table compares key aspects of mass spectrometry and affinity proteomics techniques.

Additional file 2: Table S2. Relevant blood plasma and cell type datasets.

Additional file 3: Fig. S1. Statistical comparison of PeptideAtlas protein quantification methods. Comparison of PeptideAtlas quantification metrics, raw observations and normalized values.

Additional file 4: Data S1. Proteomic Resources and Datasets Used for the Human Plasma and Cell-Type Proteome Analysis. A detailed description of the data collection process from the source databases, with links to the original databases and associated GitHub repositories provided.

Additional file 5: Fig. S2. Overlaps and differences in the identified plasma proteins of the most relevant sources used in the PaxDb database. PaxDb database is based on several datasets and PeptideAtlas versions. This plot compares them, focusing on shared and unique proteins.

Additional file 6: Data S2. Data harmonization and gene-level aggregation. Methodology used to combine and compare data from different datasets, including a link to GitHub.

Additional file 7: Data S3. Combined Circulating proteome. Methodology and curated list of the circulating proteome generated by integrating multiple databases, with a link to the GitHub repository.

Additional file 8: Data S4. PeptideAtlas and quantms complementary platform strength data. A list of Shared proteins between PeptideAtlas and quantms, divided into groups depending on their abundance and detection frequency. Includes a link to GitHub.

Additional file 9: Fig. 3S. Comparative analysis of serum proteomics databases. Comparison of serum proteome data from GPMDB and PaxDb, highlighting similarities and differences in protein representation.

Additional file 10: Data S5. Original Resource and List of Plasma Biomarkers. List of plasma biomarkers derived from MarkerDB 2.0 by selecting protein entries detected in plasma or serum, with links to the original resource and the associated GitHub repository.

Additional file 11: Data S6. List of cell subtypes investigated in the PXD004352 study. A list of cell subtypes from PXD004352, that were later combined.

Additional file 12: Table S3. Blood cell types of proteomes by combining resources. A table showing the number of proteins per cell type after the combination of several resources. The number of proteins only reported in one dataset and the overlap percentage per cell type are also included.

Additional file 13: Fig. S4. Overlaps and differences among resources on the proteome of each blood cell type. Comparison of proteins shared across databases for each cell type, shown as one UpSet plot per cell type.

Additional file 14: Data S7. Comparison between GPMDB and PXD004352 cell-type proteomes. In depth comparison and explanation of GPMDB and PXD004352 cell-type proteome discrepancies.

Additional file 15: Table S4. Overview of the overlap between the BPA and the dataset across different blood cell types. A table showing the overlap between the proteins identified plasma and cell types in this study and the Blood Proteoform Atlas.

## Data Availability

Detailed information for each database and dataset is available in Additional file 4: Data S1.
