# Supplementary material for: Blood proteomics: insights from public data
Source: Genome Biol. 2026 Mar 12;27:81. doi: 10.1186/s13059-026-04027-9 (PMC12980870; doi:10.1186/s13059-026-04027-9)
Supplement: Supplementary file 1 — Additional file 1: Table S1. Comparative overview of proteomic techniques used in blood analysis at the time of writing. This table compares key aspects of mass spectrometry and affinity proteomics techniques. [file 13059_2026_4027_MOESM1_ESM.docx]

# Additional file 1: Table S1: Comparative overview of proteomic techniques used in blood analysis at the time of writing

| **Criteria** | **Mass Spectrometry** | **Affinity proteomics** |
| --- | --- | --- |
| **Protein**  **Discovery** | **Unbiased**  No prior knowledge required | **Biased**  Relies on available antibodies |
| **Quantification** | **Absolute and relative** | **Absolute and relative** |
| **PMTs** | **Suitable**  Cover wide range and types of PTMs | **Limited**  Focused on specific PTMs |
| **Analytical**  **Sensitivity** | **High**  Specialized protocols | **Very High**  Specialized affinity binders |
| **Detection**  **Range** | **Workflow dependent**  orders of magnitude | **Panel dependent**  orders of magnitude |
| **Sample**  **Throughput** | **Moderate**  Complex sample workflow | **High**  Simplified and automated |
| **Specificity** | **High**  Depends on database matching | **Moderate**  Validation dependent |
| **Ease of Use** | **Low**  Dependent on expertise of operator | **Moderate**  Standardized kits and single protocols |
