# Supplementary material for: Blood proteomics: insights from public data
Source: Genome Biol. 2026 Mar 12;27:81. doi: 10.1186/s13059-026-04027-9 (PMC12980870; doi:10.1186/s13059-026-04027-9)
Supplement: Supplementary file 2 — Additional file 2: Table S2. Relevant blood plasma and cell type datasets. [file 13059_2026_4027_MOESM2_ESM.docx]

# Additional file 2: Table S2: Relevant blood plasma and cell type datasets.

Datasets are sorted by technique, cell type, and sample size, in that order.

| **Technique** | **Biofluid or**  **cell type** | **Sample size** | **PubMed** | **# Proteins** | **Instrument** | **Depletion,**  **Fractionation** | **Acquisition method and**  **Labelling** |
| --- | --- | --- | --- | --- | --- | --- | --- |
| **MS** | Plasma | 141 | [PMID: 32699280](https://pubmed.ncbi.nlm.nih.gov/32699280/) (2020) | 2,000+ | Orbitrap Lumos  Triple TOF 6600+ | MARS-14 and XBridge  BEH C18 column  Nanoparticles | DDA  DIA |
| **MS** | Plasma | 42 | [PMID: 30958262](https://pubmed.ncbi.nlm.nih.gov/30958262/) (2019) | 2,308 | Q-Exactive | Agilent Plasma 14 Multiple Removal System  HiRIEF | DDA  TMT-10 plex |
| **MS** | Plasma | 40 | [PMID: 39868270](https://pubmed.ncbi.nlm.nih.gov/39868270/)  (2025) | 7,000+ | Orbitrap Astral | Seer nanoparticles | DIA |
| **MS** | Plasma | 16 | [PMID: 28749931](https://pubmed.ncbi.nlm.nih.gov/28749931/)  (2017) | 4,500+ | Q Exactive Plus | Seppro IgY14 LC20 column  bRP | DDA  iTRAQ, TMT6-10 |
| **MS** | Cell type | 175 | [PMID: 28263321](https://pubmed.ncbi.nlm.nih.gov/28263321/)  (2017) | 9,500+ | Q Exactive HF | - | DDA |
| **MS** | Cell type | 156 | [PMID: 39127789](https://pubmed.ncbi.nlm.nih.gov/39127789/)  (2024) | 10,000 | Q-Exactive Orbitrap Exploris | StageTip-based high-pH | DDA  DIA |
| **MS** | Cell type | 48 | [PMID: 37388918](https://pubmed.ncbi.nlm.nih.gov/37388918/)  (2023) | 5,000+ | Q-Exactive | - | DDA |
| **MS** | Cell type | 6 | [PMID: 24870542](https://pubmed.ncbi.nlm.nih.gov/24870542/)  (2014) | 5,000-10,000 | Orbitrap Elite and Velos | In-gel digestion bRPLC | DDA |
| **ARBP** | Plasma | 38380 | [PMID: 37794188](https://pubmed.ncbi.nlm.nih.gov/37794188/)  (2023) | 2,919 | Olink Explore 3072 | - | - |
| **ARBP** | Plasma | 35559 | [PMID: 34857953](https://pubmed.ncbi.nlm.nih.gov/34857953/)  (2021) | 4,600+ | SomaScan v/4 | - | - |
| **ARBP** | Plasma | 3301 | [PMID: 29875488](https://pubmed.ncbi.nlm.nih.gov/29875488/) (2018) | 3,622 | SomaScan assay, Olink | - | - |
| **ARBP** | Plasma | 372 | [PMID: 33941778](https://pubmed.ncbi.nlm.nih.gov/33941778/)  (2021) | 1,463 | Olink Explorer | - | - |
