# Supplementary material for: Blood proteomics: insights from public data
Source: Genome Biol. 2026 Mar 12;27:81. doi: 10.1186/s13059-026-04027-9 (PMC12980870; doi:10.1186/s13059-026-04027-9)
Supplement: Supplementary file 3 — Additional file 3: Fig. S1. Statistical comparison of PeptideAtlas protein quantification methods. Comparison of PeptideAtlas quantification metrics, raw observations and normalized values. [file 13059_2026_4027_MOESM3_ESM.docx]

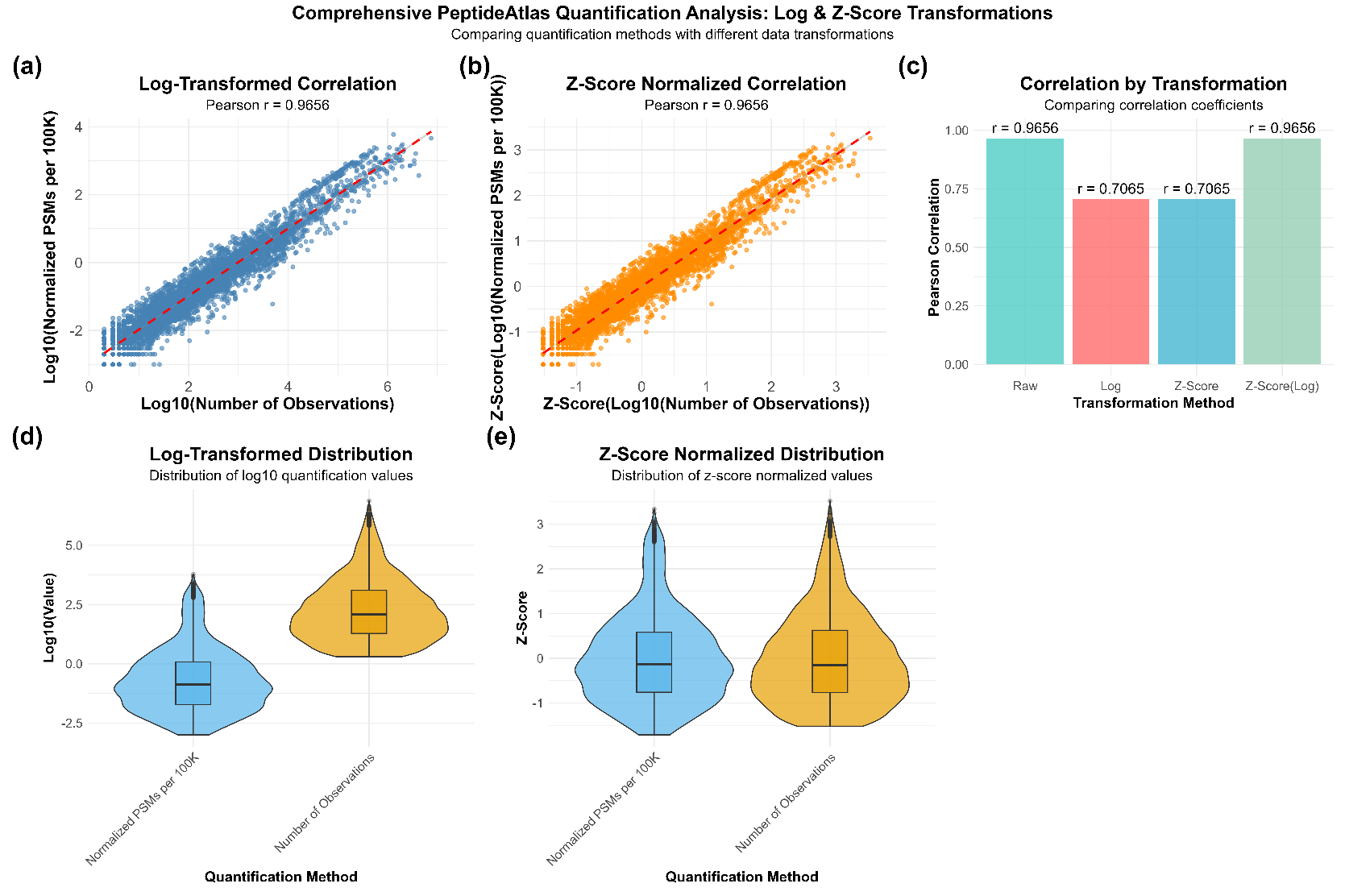


# Additional file 3: Fig. S1: Statistical comparison of PeptideAtlas protein quantification methods.

PeptideAtlas provides two protein quantification metrics: raw observation counts and normalized values ("Normalized PSM per 100K"). **A**) Log-transform correlation of the raw observations. **B**) Z-score normalized correlation of the data; **C**) Correlation score of normalization methods. **D**) Log-transform distribution of the raw observations. **E**) Z-score normalized distribution of the data.
