# Supplementary material for: Blood proteomics: insights from public data
Source: Genome Biol. 2026 Mar 12;27:81. doi: 10.1186/s13059-026-04027-9 (PMC12980870; doi:10.1186/s13059-026-04027-9)
Supplement: Supplementary file 4 — Additional file 4: Data S1. Proteomic Resources and Datasets Used for the Human Plasma and Cell-Type Proteome Analysis. A detailed description of the data collection process from the source databases, with links to the original databases and associated GitHub repositories provided. [file 13059_2026_4027_MOESM4_ESM.docx]

# Additional file 4: Data S1: Proteomic Resources and Datasets Used for the Human Plasma and Cell-Type Proteome Analysis

**PeptideAtlas database**

<https://db.systemsbiology.net/sbeams/cgi/PeptideAtlas/GetProteins?atlas_build_id=559&organism_id=2&redundancy_constraint=4&presence_level_constraint=1&action=QUERY>

PeptideAtlas data for the human plasma proteome were obtained from the Human Plasma Build (2025–08). All canonical plasma proteins were selected, and the complete dataset was downloaded for analysis.

GitHub accession: <https://github.com/asierlarrea/blood-review-data/tree/main/data/raw/peptideatlas>

**The Human Protein Atlas**

<https://www.proteinatlas.org/humanproteome/blood>

The Human Blood Atlas resource, integrated within the Human Protein Atlas database, is divided into two main sections. The first focuses on disease-related data and includes a pan-disease study where proteins are quantified using proximity extension assays (PEA) and mass spectrometry (MS). The second section is dedicated to the blood plasma proteome, presenting estimated protein concentrations in human plasma derived from both immunoassays (including PEA) and MS-based proteomics. This study only used data from this last section.

Each analytical method on the blood plasma proteome has its own dedicated page, detailing the origin and nature of the data:

- Immunoassays: Reference plasma concentrations for 308 proteins actively secreted into the blood were compiled from published studies using immunoassay techniques. They provide plasma concentrations for 308 secreted proteins, measured in studies from 1983 to 2021, range from 40 mg/mL (albumin) to 4 pg/mL (interleukin-5).
- MS: Protein concentrations were estimated using MS-based proteomics data from the publicly available PeptideAtlas Human Plasma 2025-08 build. This dataset includes estimated concentrations for 4,286 proteins, and they only provide the mean concentration for each protein. Since the most abundant proteins are usually depleted in MS assays, albumin is not present in the database, and the most abundant protein (CP) reaches 370 mg/L, while the least concentrated protein is RNF11 with 1.6 ng/L
- PEA: PEA was applied to 2,910 proteins as part of a longitudinal wellness study involving 8000 individuals over two years (with 6 measurements taken). Results are expressed as Normalized Protein eXpression (NPX) for each gene, separately for males and females. Variability in protein levels is summarized using the mean coefficient of variation (CV), calculated both across individuals at each time point and within individuals across all three visits. No concentration for each protein is provided.

GitHub accession: <https://github.com/asierlarrea/blood-review-data/tree/main/data/raw/hpa>

**PaxDb**

<https://pax-db.org/species/9606>

PaxDb data for the human plasma, serum and cell-type proteomes were obtained from the *Homo sapiens* dataset. For the plasma proteome, the integrated build was used, the PeptideAtlas 2021 build was used for serum, while the cell-type proteome data were sourced from the Kim *et al.* (2014) study.

Each dataset in PaxDb is accompanied by two key quality metrics: Coverage and the Interaction Consistency Score. Coverage indicates the proportion of the proteome detected in a dataset, with higher values reflecting greater completeness. The Interaction Consistency Score assesses quality by measuring how well abundance ratios of known interacting proteins match expected patterns, as such proteins typically show similar levels.

GitHub accession: <https://github.com/asierlarrea/blood-review-data/tree/main/data/raw/paxdb>

**GPMDB**

<https://www.thegpm.org/lists/index.html#201507081>

GPMDB data for the human plasma and cell-type proteomes were obtained from the Observed human proteins by tissue type build (2010/05/01).

GitHub accession: <https://github.com/asierlarrea/blood-review-data/tree/main/data/raw/gpmdb>

**quantms**

quantms (<https://quantms.org/datasets>) data for the human plasma proteome were obtained from the quantms platform, which provides standardized reanalysis of public mass spectrometry datasets. For this study, we retrieved the reanalyzed human plasma datasets available in quantms, ensuring consistency across identifications and quantifications through its uniform processing pipeline. Only the canonical protein groups reported in the processed output were considered for further analysis.

GitHub accession:

<https://github.com/asierlarrea/blood-review-data/tree/main/data/raw/quantms>

**PXD004352**

PXD004352 data for the human cell type proteomes were obtained from the Additional file 12: Table S3. Data from different cell subtypes, such as activated and steady state or naïve and memory B cells was combined.

GitHub accession: <http://github.com/asierlarrea/blood-review-data/tree/main/data/raw/proteomexchange>

**PXD040957**

PXD040957 data for the human cell type proteomes were obtained from the Additional file 12: Table S3 and Additional file 15: Table S4.

GitHub accession: <http://github.com/asierlarrea/blood-review-data/tree/main/data/raw/proteomexchange>
