# Supplementary material for: Blood proteomics: insights from public data
Source: Genome Biol. 2026 Mar 12;27:81. doi: 10.1186/s13059-026-04027-9 (PMC12980870; doi:10.1186/s13059-026-04027-9)
Supplement: Supplementary file 5 — Additional file 5: Fig. S2. Overlaps and differences in the identified plasma proteins of the most relevant sources used in the PaxDb database. PaxDb database is based on several datasets and PeptideAtlas versions. This plot compares them, focusing on shared and unique proteins. [file 13059_2026_4027_MOESM5_ESM.docx]

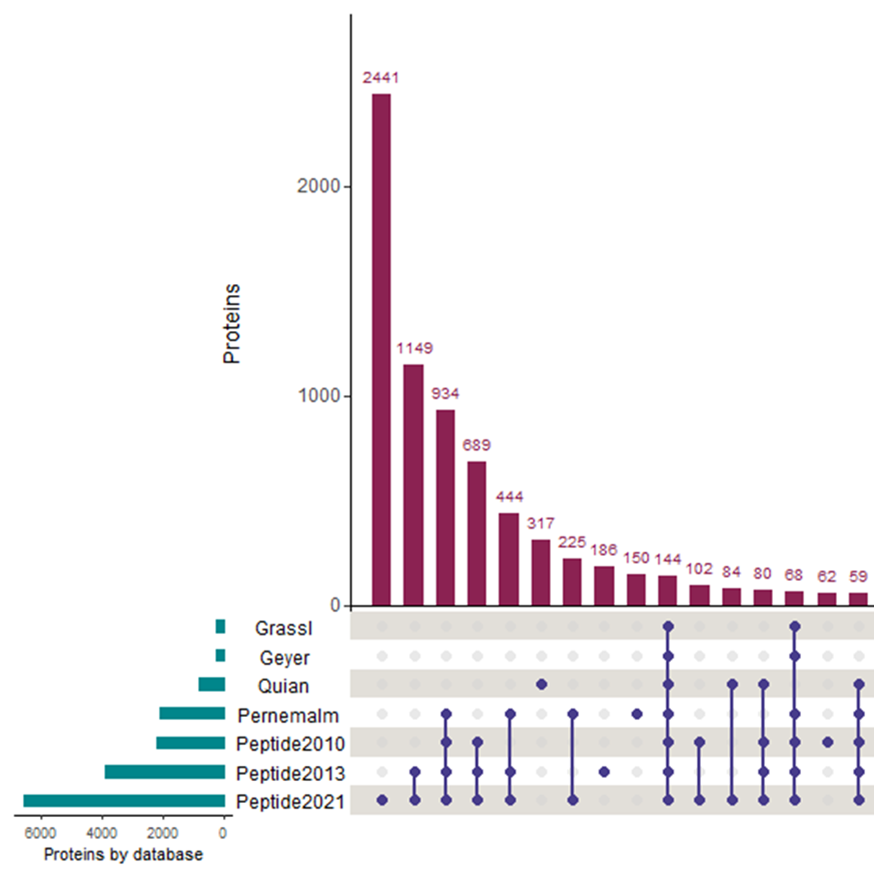


# Additional file 5: Fig. S2: Overlaps and differences in the identified plasma proteins of the most relevant sources used in the PaxDb database.

Each set in the graph represents a unique combination of identified proteins, while the intersections indicate shared elements among the databases. The smaller bars to the left indicate the number of identified proteins per database. Combinations with less than 50 proteins are not shown.
