# Supplementary material for: Blood proteomics: insights from public data
Source: Genome Biol. 2026 Mar 12;27:81. doi: 10.1186/s13059-026-04027-9 (PMC12980870; doi:10.1186/s13059-026-04027-9)
Supplement: Supplementary file 6 — Additional file 6: Data S2. Data harmonization and gene-level aggregation. Methodology used to combine and compare data from different datasets, including a link to GitHub. [file 13059_2026_4027_MOESM6_ESM.docx]

# Additional file 6: Data S2: Data harmonization and gene-level aggregation.

To enable consistent comparison of plasma proteome data across multiple public resources, we developed a standardized processing pipeline involving normalization, identifier harmonization, and gene-level aggregation. Protein abundance values were retrieved from various databases, including quantms, PeptideAtlas, ProteomeXchange, HPA, PaxDb, and GPMDB. These sources report different types of measurements, such as iBAQ (quantms), spectral counts (GPMDB), PSMs per 100,000 spectra (PeptideAtlas), parts per million (PaxDb), and NPX or mg/L (HPA). To account for the differing value distributions and measurement scales across sources, we applied a robust quantile-to-normal transformation. Within each database, protein abundance values were first log-transformed and ranked to obtain quantile scores, which were then converted into standard normal deviates (z-scores) using the inverse normal cumulative distribution function. This approach preserves relative ranking while aligning data distributions across datasets. All protein identifiers were mapped to HGNC gene symbols using a cached mapping system that supports both UniProt and Ensembl accessions. The mapping achieved a 96% success rate and used fallback strategies to resolve ambiguous or missing entries. In cases where a single protein group mapped to multiple gene symbols, the data were excluded to maintain specificity. After mapping, redundant protein measurements corresponding to the same gene within each dataset—such as multiple isoforms or technical replicates—were aggregated by taking the median abundance value. This deduplication step ensured that the final dataset reflected unambiguous, non-redundant gene-level representations. The result is a harmonized list of plasma proteins expressed as normalized gene-level intensities, suitable for integrative analysis across heterogeneous proteomics platforms. All downstream analyses and visualizations were therefore based on gene-level protein abundance.

GitHub accession: <https://github.com/asierlarrea/blood-review-data/blob/main/README.md>
