# Supplementary material for: Blood proteomics: insights from public data
Source: Genome Biol. 2026 Mar 12;27:81. doi: 10.1186/s13059-026-04027-9 (PMC12980870; doi:10.1186/s13059-026-04027-9)
Supplement: Supplementary file 7 — Additional file 7: Data S3. Combined Circulating proteome. Methodology and curated list of the circulating proteome generated by integrating multiple databases, with a link to the GitHub repository. [file 13059_2026_4027_MOESM7_ESM.docx]

# Additional file 7: Data S3: Combined circulating proteome.

The combined circulating proteome was obtained by combining PeptideAtlas, GPMDB, PaxDb, quantms, and the HPA, encompassing MS and PEA.

GitHub accession:

[https://github.com/asierlarrea/blood-review-data/tree/main/outputs/tables/01_plasma_protein_analysis/proteins_presence_summary.txt](https://github.com/asierlarrea/blood-review-data/tree/main/outputs/tables/01_plasma_protein_analysis/proteins_presence_summary.csv)
