# Supplementary material for: Blood proteomics: insights from public data
Source: Genome Biol. 2026 Mar 12;27:81. doi: 10.1186/s13059-026-04027-9 (PMC12980870; doi:10.1186/s13059-026-04027-9)
Supplement: Supplementary file 8 — Additional file 8: Data S4. PeptideAtlas and quantms complementary platform strength data. A list of Shared proteins between PeptideAtlas and quantms, divided into groups depending on their abundance and detection frequency. Includes a link to GitHub. [file 13059_2026_4027_MOESM8_ESM.docx]

# Additional file 8: Data S4: PeptideAtlas and quantms complementary platform strength data.

Shared proteins between PeptideAtlas and quantms were divided into groups depending on their abundance and detection frequency.

GitHub accession:

[https://github.com/asierlarrea/blood-review-data/tree/main/outputs/tables/01_plasma_protein_analysis/proteins_peptideatlas_vs_quantms_groups.txt](https://github.com/asierlarrea/blood-review-data/tree/main/outputs/tables/01_plasma_protein_analysis/proteins_presence_summary.csv)
