# Supplementary material for: Blood proteomics: insights from public data
Source: Genome Biol. 2026 Mar 12;27:81. doi: 10.1186/s13059-026-04027-9 (PMC12980870; doi:10.1186/s13059-026-04027-9)
Supplement: Supplementary file 9 — Additional file 9: Fig. 3S. Comparative analysis of serum proteomics databases. Comparison of serum proteome data from GPMDB and PaxDb, highlighting similarities and differences in protein representation. [file 13059_2026_4027_MOESM9_ESM.docx]

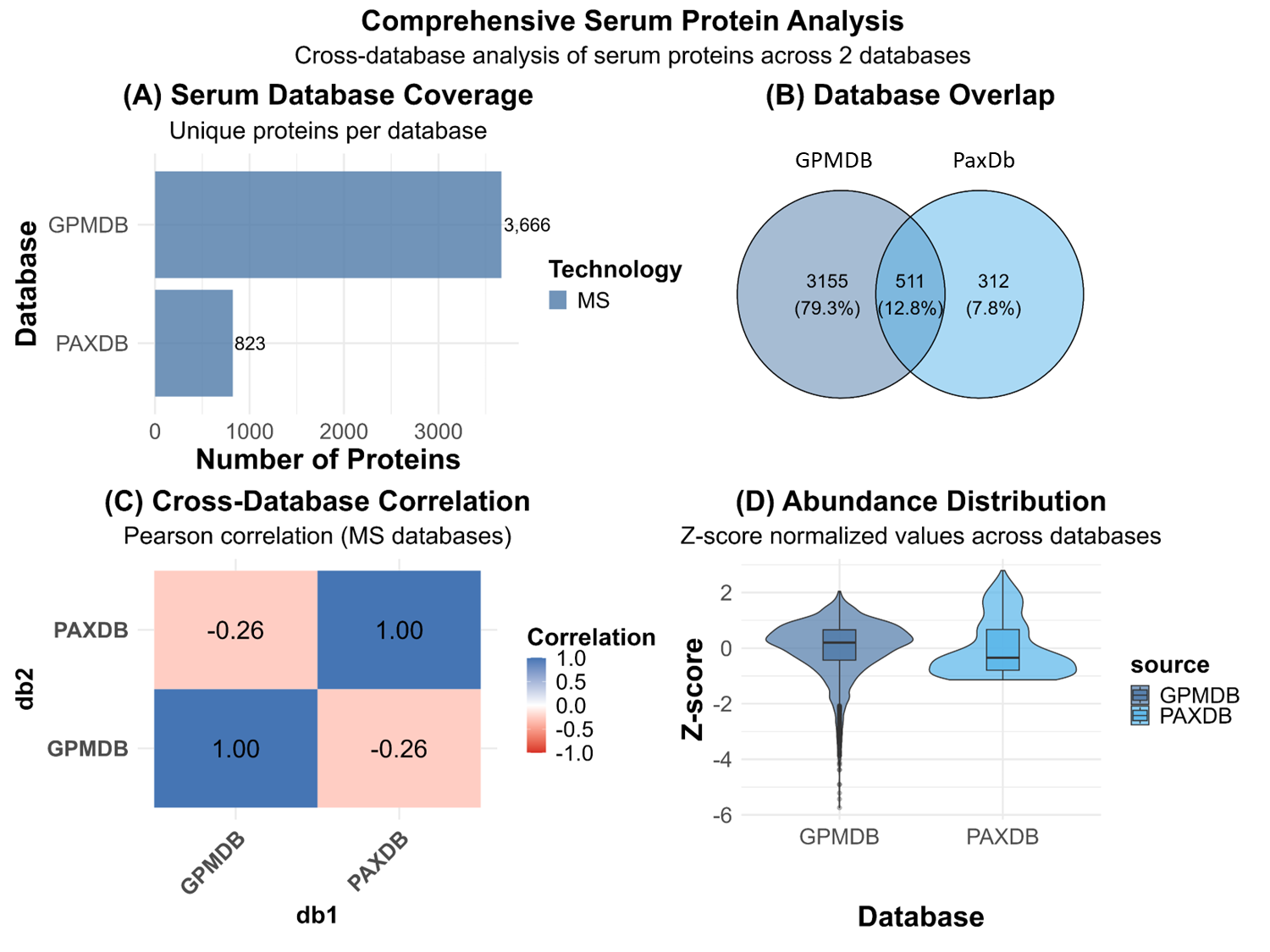


# Additional file 9: Fig. 3S: Comparative analysis of serum proteomics databases.

**(A)** Number of proteins identified in each database (GPMDB and PaxDb). Counts reflect the number of unique genes detected in each source. **(B)** Cross-database correlation of PaxDb and GPMDB after Z-score normalization. Values are standardized within each dataset and do not reflect absolute concentrations. Pearson correlation is calculated pairwise for shared proteins. **(C)** Distribution of protein abundance values in each database after Z-score normalization. Values are scaled to mean 0 and standard deviation 1 within each database. **(D)** Violin and boxplot representation of Z-score normalized protein abundances, illustrating distribution and spread across databases.
