# Supplementary material for: Blood proteomics: insights from public data
Source: Genome Biol. 2026 Mar 12;27:81. doi: 10.1186/s13059-026-04027-9 (PMC12980870; doi:10.1186/s13059-026-04027-9)
Supplement: Supplementary file 10 — Additional file 10: Data S5. Original Resource and List of Plasma Biomarkers. List of plasma biomarkers derived from MarkerDB 2.0 by selecting protein entries detected in plasma or serum, with links to the original resource and the associated GitHub repository. [file 13059_2026_4027_MOESM10_ESM.docx]

# Additional file 10: Data S5: Original Resource and List of Plasma Biomarkers.

The list of plasma biomarkers was obtained from MarkerDB 2.0 (<https://markerdb.ca/downloads>). From the available datasets, the **Protein** table was selected, and entries were filtered based on the “biofluid” column to include only proteins detected in plasma or serum.

GitHub accession: <https://github.com/asierlarrea/blood-review-data/blob/main/data/metadata/biomarkers_list.csv>
