# Supplementary material for: Blood proteomics: insights from public data
Source: Genome Biol. 2026 Mar 12;27:81. doi: 10.1186/s13059-026-04027-9 (PMC12980870; doi:10.1186/s13059-026-04027-9)
Supplement: Supplementary file 11 — Additional file 11: Data S6. List of cell subtypes investigated in the PXD004352 study. A list of cell subtypes from PXD004352, that were later combined. [file 13059_2026_4027_MOESM11_ESM.docx]

# Additional file 11: Data S6: List of cell subtypes investigated in the PXD004352 study.

T4_naive_, T4_TCM_, T4_TEM_, T4_TEMRA_, T_reg naive_, T_reg memory_, T4_TH1_, T4_TH2_, T4_TH17_, T8_naive_, T8_TCM_, T8_TEM_, T8_TEMRA_, NK_CD56 bright_, NK_CD56 dim_, B_naive_, B_memory_, B_plasma_, MO_classical_, MO_nonclassical_, MO_intermediate_, DC_CD1c_, DC _CD304_, Neutrophils, Eosinophils, Basophil.
