# Supplementary material for: Blood proteomics: insights from public data
Source: Genome Biol. 2026 Mar 12;27:81. doi: 10.1186/s13059-026-04027-9 (PMC12980870; doi:10.1186/s13059-026-04027-9)
Supplement: Supplementary file 12 — Additional file 12: Table S3. Blood cell types of proteomes by combining resources. A table showing the number of proteins per cell type after the combination of several resources. The number of proteins only reported in one dataset and the overlap percentage per cell type are also included. [file 13059_2026_4027_MOESM12_ESM.docx]

# Additional file 12: Table S3: Blood cell types of proteomes by combining resources.

Combined reported proteins, proteins reported in one or all resources, and unique proteins for each cell type are detailed. Reported proteins refer to those listed across sources but do not imply confirmed identification; they may include unverified or context-dependent entries.

| **Cell type** | **Combined reported proteins** | **Proteins reported in only one resource** | **Overlapped protein among all resources** |
| --- | --- | --- | --- |
| **CD8+** | 13053 | 4755 (36%) | (28%) |
| **CD4+** | 12573 | 6064 (48%) | 1278 (31%) |
| **B cell** | 10864 | 4587 (42%) | 6277 (58%) |
| **NK** | 11187 | 5729 (52%) | 5458 (48%) |
| **Platelet** | 5713 | 4743 (83%) | 970 (17%) |
| **Erythrocyte** | 846 | 842 (99%) | 4 (1%) |
| **Monocyte** | 9549 | 5731 (60%) | 3818 (40%) |
| **DC** | 7735 | - | - |
| **Macrophage** | 6328 | - | - |
| **Neutrophil** | 6742 | - | - |
| **Eosinophil** | 7222 | - | - |
| **Basophil** | 7631 | - | - |
