# Supplementary material for: Blood proteomics: insights from public data
Source: Genome Biol. 2026 Mar 12;27:81. doi: 10.1186/s13059-026-04027-9 (PMC12980870; doi:10.1186/s13059-026-04027-9)
Supplement: Supplementary file 13 — Additional file 13: Fig. S4. Overlaps and differences among resources on the proteome of each blood cell type. Comparison of proteins shared across databases for each cell type, shown as one UpSet plot per cell type. [file 13059_2026_4027_MOESM13_ESM.docx]

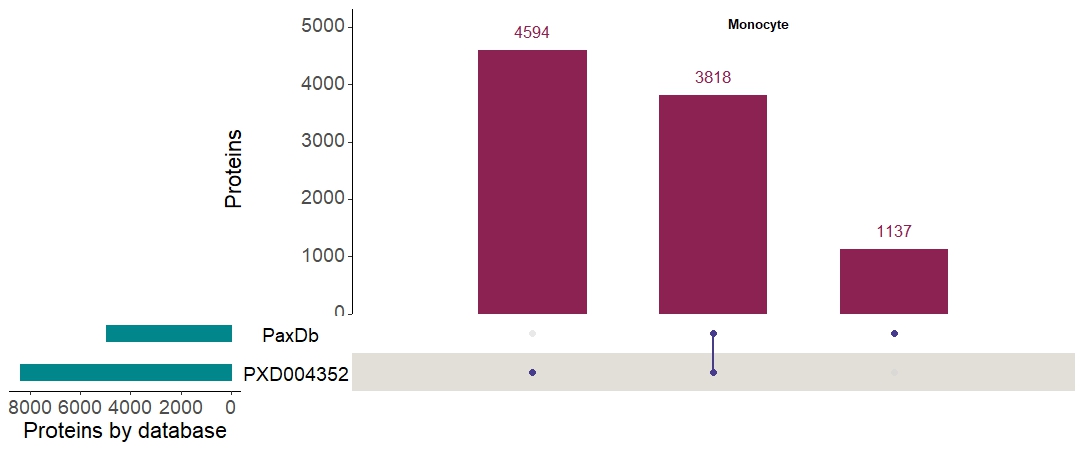

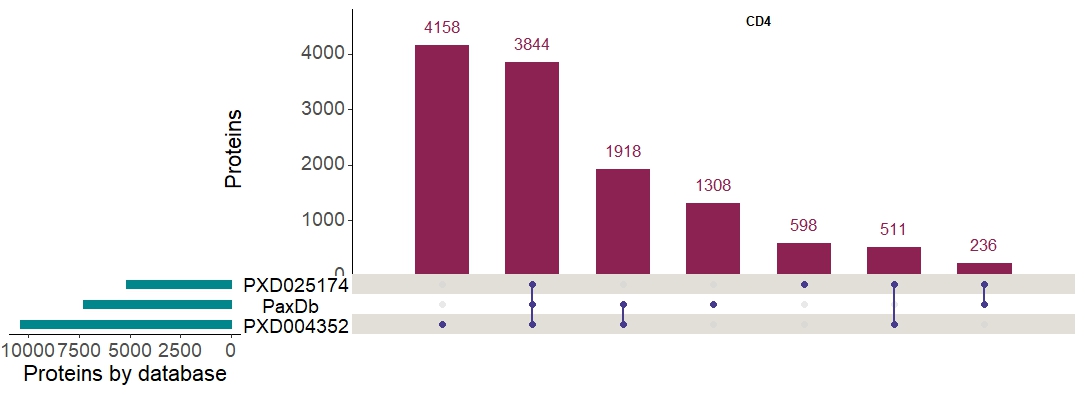

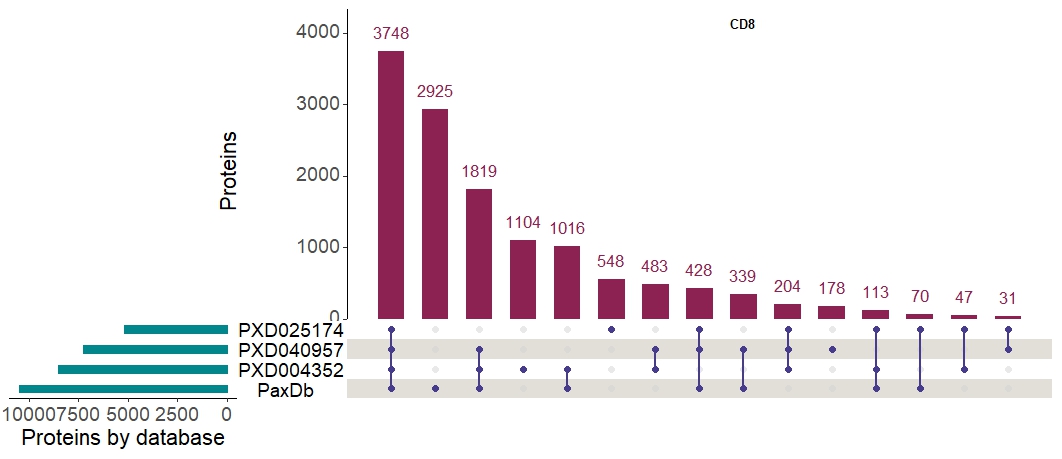


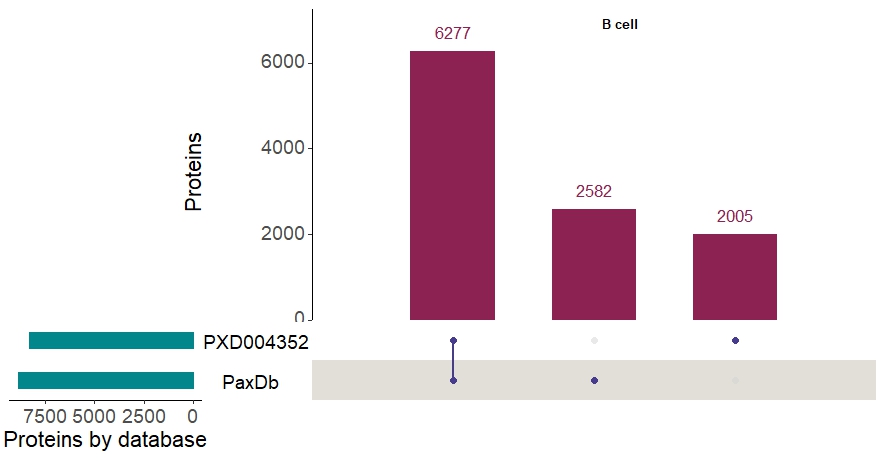


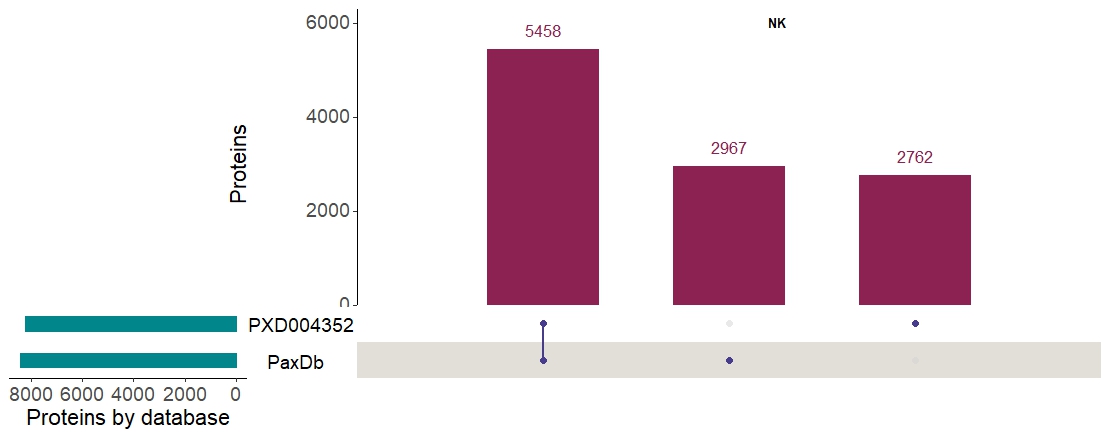


# Additional file 13: Fig. S4: Overlaps and differences among resources on the proteome of each blood cell type.

Each set in the graph represents a unique combination of identified proteins, while the intersections indicate shared elements among the databases. The smaller bars to the left indicate the number of identified proteins per database. The submissions refer to: (i) the proteomic characterization of conserved TCR signalling complexes in primary human CD4⁺ and CD8⁺ T cells (PXD025174); (ii) the proteomic profiling of blood- and tumor-derived immune cell types in liver cancer to uncover mechanisms of immune dysfunction (PXD040957); and (iii) a comprehensive proteomic atlas of primary human blood immune cell types and their communication networks (PXD004352).
