# Supplementary material for: Blood proteomics: insights from public data
Source: Genome Biol. 2026 Mar 12;27:81. doi: 10.1186/s13059-026-04027-9 (PMC12980870; doi:10.1186/s13059-026-04027-9)
Supplement: Supplementary file 14 — Additional file 14: Data S7. Comparison between GPMDB and PXD004352 cell-type proteomes. In depth comparison and explanation of GPMDB and PXD004352 cell-type proteome discrepancies. [file 13059_2026_4027_MOESM14_ESM.docx]

# Additional file 14: Data S7: Comparison between GPMDB and PXD004352 cell-type proteomes

Comparison of cell-type proteomes between GPMDB and PXD004352 revealed notable discrepancies for platelets and erythrocytes, with only 17% and 1% of their combined proteomes shared, respectively. Protein concentration changes generally showed moderate correlations (0.5–0.7) across database pairs, but the GPMDB–PXD004352 comparisons for erythrocytes and platelets were the only cases below 0.5 (0.08 for erythrocytes, 0.36 for platelets).

The low concordance between GPMDB and PXD004352 for erythrocytes and platelets likely reflects the age and heterogeneous nature of GPMDB’s cell-type submissions. As an older resource, GPMDB aggregates datasets from multiple studies with diverse sample preparation protocols, variable MS platforms, and often incomplete or inconsistent metadata. These factors make direct harmonization with more recent, well-annotated datasets challenging, and suggest that the low overlaps and correlation values observed are due to database heterogeneity rather than true biological variation
