# Supplementary material for: Blood proteomics: insights from public data
Source: Genome Biol. 2026 Mar 12;27:81. doi: 10.1186/s13059-026-04027-9 (PMC12980870; doi:10.1186/s13059-026-04027-9)
Supplement: Supplementary file 15 — Additional file 15: Table S4. Overview of the overlap between the BPA and the dataset across different blood cell types. A table showing the overlap between the proteins identified plasma and cell types in this study and the Blood Proteoform Atlas. [file 13059_2026_4027_MOESM15_ESM.docx]

# Additional file 15: Table S4: Overview of the overlap between the BPA and the dataset across different blood cell types.

The table highlights the extent to which proteins identified in the BPA are also present in our data, as well as the proportion they represent within the full proteome we observed for each cell type. This comparison illustrates both the high concordance between the datasets and the limited coverage of the BPA relative to our broader proteomic analysis.

| **Cell type** | **Unique Genes** | **Overlap with this study** | **Overlap with this study (%)** | **Total genes in this study** | **Total genes in this study (%)** |
| --- | --- | --- | --- | --- | --- |
| **Erythrocyte** | 129 | 19 | 14,7 | 846 | 2,2 |
| **Platele** | 352 | 275 | 78,1 | 5713 | 4,81 |
| **Macrophage** | 556 | 484 | 87,0 | 6327 | 7,65 |
| **B cell** | 793 | 768 | 96,8 | 10864 | 7,07 |
| **DC** | 695 | 547 | 78,7 | 7735 | 7,07 |
| **Monocyte** | 694 | 667 | 96,1 | 9549 | 6,99 |
| **NK** | 758 | 729 | 96,1 | 11187 | 6,52 |
| **Eosinophil** | 450 | 319 | 70,8 | 7222 | 4,42 |
| **Neutrophil** | 421 | 306 | 72,6 | 6741 | 4,54 |
| **CD4** | 673 | 652 | 96,8 | 12573 | 5,19 |
| **CD8** | 552 | 537 | 97,2 | 13053 | 4,11 |
| **Plasma** | 86 | 85 | 98,8 | 8038 | 1,06 |
